# Effects of Peripheral Cooling on Upper Limb Tremor Severity and Functional Capacity in Persons with MS

**DOI:** 10.3390/jcm12134549

**Published:** 2023-07-07

**Authors:** Peter Feys, Marijke Duportail, Daphne Kos, Stephan Ilsbroukx, Ilse Lamers, Paul Van Asch, Werner Helsen, Lousin Moumdjian

**Affiliations:** 1 REVAL Rehabilitation Research Center, Research Institute, Faculty of Rehabilitation Sciences, Hasselt University, 3590 Diepenbeek, Belgium; ilse.lamers@uhasselt.be (I.L.); lousin.moumdjian@uhasselt.be (L.M.); 2 University MS Centre Hasselt-Pelt, 3500 Hasselt, Belgium; 3 National MS Center Melsbroek, 1820 Steenokkerzeel, Belgiumdaphne.kos@kuleuven.be (D.K.);; 4 Faculty of Kinesiology and Rehabilitation Sciences FABER, Katholieke Universiteit Leuven, 3001 Leuven, Belgium; werner.helsen@kuleuven.be; 5 Rehabilitation and MS Center Noorderhart, 3900 Pelt, Belgium; 6 Fitness and Physiotherapy Center, 2550 Kontich, Belgium; paulvanasch@yahoo.com

**Keywords:** intention tremor, cooling, upper limb, functional performance, Fahn tremor rating scale, multiple sclerosis

## Abstract

Upper limb intention tremor in persons with multiple sclerosis (pwMS) affects the ability to perform activities of daily life and is difficult to treat. The study investigated the effect of peripheral upper limb cooling on tremor severity and functional performance in MS patients with intention tremor. In experiment 1, 17 patients underwent two 15 min cooling conditions for the forearm (cold pack and cryomanchet) and one control condition. In experiment 2, 22 patients underwent whole arm cooling for 15 min using multiple cold packs. In both experiments, patients were tested at four time points (pre- and post-0, -25 and -50 min cooling) on unilateral tasks of the Test Evaluant les Membres supérieurs des Personnes Agées (TEMPA), Fahn’s tremor rating scale (FTRS), Nine Hole Peg Test (NHPT). In experiment 1, the mean FTRS ranged from 13.2 to 14.1 across conditions. A two-way ANOVA showed mainly time effects, showing that cooling the forearm significantly reduced the FTRS, the performance on the NHPT, and three out of four items of the TEMPA, mostly independent of the cooling modality. In experiment 2, the mean FTRS was 13.1. A repeated measures ANOVA showed that cooling the whole arm reduced the FTRS and time needed to execute two out of four items of the TEMPA. These effects occurred immediately after cooling lasting at least 25 min. Cooling the whole upper limb led to a clinically noticeable effect on tremor severity and improved functional performance, which was pronounced during the first half-hour after cooling.

## 1. Introduction

Tremor, the involuntary rhythmic oscillatory movement of a body part, is estimated to occur in 20 to 60 percent of patients diagnosed with multiple sclerosis (MS). The tremor of multiple sclerosis is frequently embedded in a complex movement disorder, which often includes dysmetria and other ataxic features [1,2,3]. Tremor in MS can involve the upper limbs, head, lower limbs, vocal cords, and trunk. In MS, the two most prevalent tremor forms are postural tremor, tremor present whilst voluntarily maintaining a position against gravity, and intention tremor (or goal-directed tremor), defined as an increase in tremor amplitude toward the termination of a visually guided goal-directed movement of the upper limb [1,4]. The prevalence of intention tremor in the upper extremity is estimated to be one-third in MS patients [5]. Intention tremor is often used synonymously with cerebellar tremor, which is diagnosed according to the following clinical signs: pure or dominant intention tremor, uni- or bi-lateral; tremor frequency generally below 5 Hz; and the possible presence of postural tremor, but not of rest tremor [3]. Intention tremor in MS is primarily caused by lesions in the afferent and/or efferent pathways of the cerebellum [6,7].

Tremor of the upper limbs can be very disabling and seriously impair activities of daily living and quality of life [2,8]. MS patients with intention tremor show considerable difficulties in performing goal-directed functional tasks, such as drinking, writing, and using a computer mouse [6,7]. Persons with tremor reported that they felt disabled, either because of the physical effects of tremor, because they were embarrassed by the tremor, or both. Tremor-related disability is strongly correlated with overall upper limb tremor severity [1].

The treatment of tremor remains a great challenge. Medical treatment is limited in its efficacy, as the reporting of the beneficial effect of drugs has been inconsistent [9,10]. The surgical treatment options for tremor in MS are stereotactic thalamotomy and deep brain stimulation (DBS) [9,11], but these are only considered for patients with very severe tremor with inconsistent long-term and functional outcomes.

Physiotherapeutic and rehabilitation approaches such as weighted wrist cuffs and limb cooling have been applied in MS to improve daily living activities in addition to compensatory aids or techniques [2,12]. Weighted wrist cuffs are a mechanical tool to reduce tremor amplitude; however, their effect on intention tremor was found to be small or potentially not present [2,8,13] Several studies revealed that cooling the arm can have a positive effect on tremor in multiple sclerosis. Albrecht et al. (1998) [14] cooled the forearm, including the hand, by dipping it into a bath of ice for one minute. Clinically rated tremor reduction and an improvement on several clinical tests were reported between 15 and 45 min after the cooling intervention. The same procedure was applied in the study of Quintern et al. (1998) [15], in which the cooling of the whole arm led to a decrease in postural tremor, but not in objectively measured intention tremor during a pointing movement. In a previous study from our group (2005) [16], a moderate (25 °C) and a deep cooling intervention (18 °C) were applied for 15 min to the forearm, with the exclusion of the hand and the wrist joint. This was carried out using a cryomanchet connected to an active cooling system, with the aim of examining the effects on intention tremor in MS patients. Objective recordings taken during a wrist step tracking task showed that the overall amplitude and frequency of tremor reduced immediately after moderate cooling, and even more after deep cooling. This dosage-related effect remained present during the full post-cooling period of 30 min. The cooling-induced tremor reduction may be explained by a combination of decreased nerve conduction velocity, changed muscle contraction properties, and reduced muscle spindle activity, which is temperature-dependent. The latter study did not, however, investigate whether or not the tremor reduction also led to the improved functionality of the upper extremity. 

In the present study, two sequential experiments were performed to investigate the effect of upper limb cooling on reducing tremor severity and improving functional capacity in MS patients with intention tremor. In experiment 1, the forearm was cooled for 15 min. Three different conditions were distinguished and applied to all participants: two cooling conditions (cold pack and cryomanchet) and one control condition. In experiment 2, performed on a different sample, the whole arm was cooled for 15 min, using multiple cold packs. In both the experiments, patients were tested four times (pre- and post-0′, post-25′ and post-50′) to evaluate whether or not cooling had an impact on tremor severity and arm functionality. 

## 2. Materials and Methods

**Figure 1:**
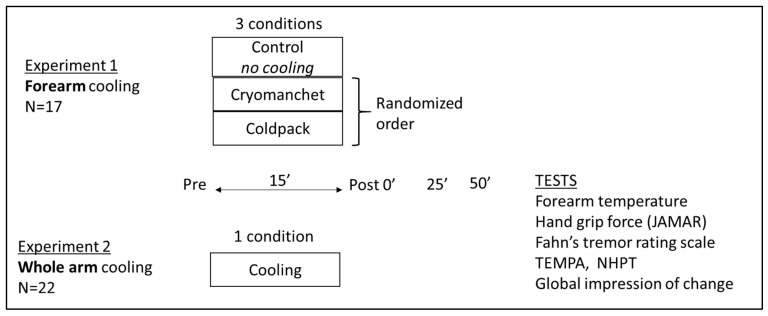
Overview of the sample size, conditions, time points, and tests for experiment 1 (N = 17) and 2 (N = 22). The global impression of change was applied in experiment 2 only.

Below, the two experiments conducted are described. An overview is also provided in Figure 1.

### 2.1. Participants 

In experiment 1, 17 MS patients with upper limb intention tremor (nine men and eight women; mean age, 46.4 years; range, 33–57 years) were selected from the National MS Center in Melsbroek. In experiment 2, 22 patients with intention tremor (10 men and 12 women; mean age, 51.1 years; range, 34–75 years) were selected from the National MS Center in Melsbroek (*n* = 19) and Rehabilitation and MS Center in Noorderhart, Overpelt (*n* = 3). Participants showing, in the upper limb, ng spasticity, muscle paresis, or sensory loss, or patients with a relapse one month before participation were not included. Tests were preferably performed with the dominant arm. The non-dominant arm was used if the tremor in the dominant arm was absent or too severe. The study was approved by the ethics committee of the University of Leuven and the local ethics committee of both clinical centers. Informed consent was obtained from all participants. Table 1 summarizes the clinical characteristics of the patient groups.

### 2.2. Experimental Procedure

#### 2.2.1. Clinical Assessment

The assessment was similar in both experiments. Grip force (kg) was measured using a handheld dynamometer (JAMAR^®^, JA Preston CO, Jackson, MI, USA) [17]. The Fahn tremor rating scale (FTRS) was used to assess the degree of intention tremor and to evaluate performance during functional tasks [18,19,20]. The degree of tremor severity was scored for every item of the FTRS, on an ordinal scale ranging from 0 (no tremor) to 4 (severe tremor). The total score of the FTRS, and two sub-scores, namely part A (items “finger nose”, “postural extended” and “postural flexed”) and part B (items “pouring water”, “writing” and “spirography”), were measured. This instrument has very good inter-rater and intra-rater reliability. The Nine Hole Peg Test (NHPT) was used to evaluate hand dexterity [21]. The score represents the total time (in seconds) needed to put nine pegs into nine holes and remove them one by one. The time limit was 300 s. The ‘Test Evaluant les Membres supérieurs des Personnes Agées’ (TEMPA) was used to measure the functional performance of the upper limbs [22]. The four unilateral tasks (“pick up and move a jar”; “pick up a pitcher and pour water into a glass”; “handle coins”; “pick up and move small objects”) were applied [23]. The test has been validated before in MS patients with an upper extremity dysfunction due to muscle weakness or intention tremor [23].

The performance on every item of the TEMPA was scored in two ways; one score represented the time needed to perform the task (expressed in seconds) and the other was a functional score (0 to −3), indicating to which degree the patient could execute the task: completely (score 0), after adaptations (score −1, −2) or not (score −3). 

In addition, in experiment 2, the patients were also asked to evaluate how difficult it was to accomplish all the given tasks and how many tremors they experienced on the visual analogue scale (VAS) (ranging from 0 to 10 cm). Post-0′, the Patient’s Global Impression of Change (PGIC), also known as the Clinical Global Impression of Change (CGIC), was used to determine the patients’ opinion about changes in tremor. Patients could determine on an ordinal scale ranging from −3 (much, much worse) to 3 (much, much better) whether or not and how they experienced a change in tremor immediately after cooling.

#### 2.2.2. Cooling Modalities in Experiments 1 and 2

Experiment 1: forearm cooling

Two different cooling conditions were distinguished, and applied on all participants in a randomized order. One cooling condition consisted of the application of a cryomanchet (23/34 cm) to the forearm, which is identical to the conditions of a previous study [17]. A cooling fluid continuously circulated through the cryomanchet from an active cooling device (EMC Medical Instruments, Maaseik, Belgium) that allowed the precise regulation of the fluid’s temperature (experiment 1). The other cooling condition consisted of applying a cold pack (28/36 cm) to the forearm for 15 min (experiment 1). The temperature of the forearm was continuously measured during the test period.

Experiment 2: whole arm cooling

The cooling condition consisted of applying multiple cold packs to the whole arm for 15 min. The arm was protected from freezing wounds and skin irritation by placing paper tissues between the cold pack and the skin of the arm. Patients were asked to rate the occurrence of pain during cooling using a VAS. The temperatures of the shoulder, elbow, and wrist were continuously measured during the test period. The temperature of the shoulder was evaluated with an infrared thermometer (5 cm distal to the shoulder joint, measured laterally), while the temperatures of the elbow and the wrist were continuously measured with two temperature probes (Fluke, Everett, WA, USA), one attached to the dorsal side of the wrist and one 3 cm distal to the elbow joint. 

For both experiments, the room temperature was kept as stable as possible during the whole test procedure to avoid influencing the test results.

### 2.3. Experimental Design and Statistical Analyses

Both experiments were characterized via repeated clinical evaluation being before and up to 50 min after cooling with time intervals of 25 min (pre, and post-0′, post-25′, and post-50′). At each test time point, the skin temperatures were registered while the clinical assessment was performed in a random order. During a control condition without cooling (see infra for experiment 1), four test blocks were distinguished with a rest period in between.

#### 2.3.1. Experiment 1: Forearm Cooling

Subjects performed the three test sessions, on separate days, at approximately the same time of day. A control condition without cooling was applied first to investigate the possible fatigue effects in MS patients with tremor in repeatedly executing the clinical assessment. Two cooling conditions consisted of forearm cooling using the cryomanchet or cold pack. Conditions were applied in random order.

Data from 17 patients were collected. The control condition was performed on 16 patients, since this condition could not be applied to one patient within his hospitalization period. The cold pack and cryomanchet conditions were applied to 16 patients, as one patient refused any cooling intervention. As such, the results of 16 patients were analyzed for each condition. A repeated measures ANOVA (pre-, post-0′, post-25′, and post-50′) for each condition was conducted for 8 variables (forearm temperature, grip force, total score of Fahn’s tremor rating scale, four items of the TEMPA, and NHPT), for both the control and cooling conditions. Bonferroni post hoc tests were used to correct for multiple comparisons. To compare the differential effects of the two cooling modalities on the items that were significantly influenced by both cooling interventions, a two-way (Cooling condition × time) ANOVA was performed. The level of significance was set at *p* < 0.05.

#### 2.3.2. Experiment 2: Whole-Arm Cooling

Twenty-two patients participated in the study; however, three patients were withdrawn for the data analysis. One patient suffered from fatigue that interfered with test performance at various time points, and the other two patients had an extremely great tremor that made it impossible to perform the majority of the tasks and complete the whole test procedure within the allotted time. Therefore, the results of 19 patients were analyzed.

A repeated measures ANOVA (pre-, and post-0′, post-25′, and post-50′) was conducted for the following variables: shoulder, elbow, and wrist temperature, grip force, the FTRS, four timed items of the TEMPA, NHPT, VAS 1, and VAS 2. The Bonferroni post hoc tests were used to correct for multiple comparisons. The level of significance was set at *p* < 0.05. All patient data were analyzed using the statistical methods described above, but the data of patients who could not perform the NHPT or an item of the TEMPA (functional score of −3) in the pre-condition were not included for further analysis, even when they completed the items during later test procedures (post-0′, post-25, and post-50′). This restriction resulted in a reduced statistical analyzed sample of 14 patients for the item “pick up a pitcher and pour water into a glass”, 17 patients for the item “handle coins”, 18 patients for the item “pick up and move small objects” of the TEMPA and 14 patients for the NHPT. Further analyses of subgroups were conducted to determine if a differential effect of cooling in patients with severe tremor exists, compared to that in patients with mild to moderate tremor. The median total score of the Fahn tremor rating scale at the time before measurement—i.e., 12—was used to distinguish between the groups. Eight patients were part of the subgroup who had fewer tremors, while 11 patients belonged to the subgroup with more tremors. Separate two-way ANOVAs were performed for these parametric variables that were significantly altered by cooling, to investigate interactions between subgroups (more or less tremor) and time points (pre-, and post-0′, post-25′, and post-50′).

## 3. Results

### 3.1. Experiment 1: Forearm Cooling 

Table 2 provides an overview of the results in the control, cold pack and cryomanchet condition (*n* = 16). No baseline (pre-measurement) differences between groups were found, indicating that patients performed similarly across multiple test moments. In the control condition, no significant performance differences were observed during the repeated executions, indicating that fatigue or practice did not influence the performance positively or negatively. 

The results of the two cooling conditions in experiment 1 will be described collectively.

#### 3.1.1. Skin Temperature

The skin temperature on the forearm differed significantly between time points (F(3.45) = 265.0; *p* < 0.0001). The temperature was significantly reduced immediately after cooling (on average by 15.2 °C at the forearm). Warm-up after removal of the cryomanchet or the cold pack was significant and seemed to happen in a curvilinear way, with the fastest recuperation occurring during the first 25 min. However, normalization of the forearm skin temperature did not take place within 50 min. No significant interaction effects were observed for the skin temperature of the forearm, indicating a similar cooling effect of both the cryomanchet and cold pack on skin temperature. 

#### 3.1.2. Handgrip Force and FTRS

Cooling with a cold pack and cryomanchet did not significantly change the handgrip force. Cooling the forearm using a cold pack or a cryomanchet significantly reduced the total scores in part A and part B, and the total score of the FTRS. The total FTRS score was significantly reduced (F(3.87) = 27.75; *p* < 0.0001), and this occurred up to 50 min after cooling. However, compared to immediately after cooling, the FTRS total was borderline significant 25′ after cooling (*p* = 0.05) and significantly greater post-50′ cooling (*p* < 0.0001). The time x group interaction (F(3.87) = 7.86; *p* < 0.001) indicated that cooling with a cryomanchet induced a larger decrease in the total FTRS score, immediately after the cooling period compared to that after cooling with a cold pack. Similarly, a significant time effect was found for part A of the FTRS (F(3.87) = 14.85; *p* < 0.0001). Post hoc tests revealed a significant decrease in tremor severity between the pre-measurement and immediately (post-0) and 25′ after cooling (*p* < 0.0001), as well as between the post-0 and post-25′, and post-0 and post-50′ timepoints (*p* < 0.001). A significant time x group interaction was also found (F(3.87) = 2.73; *p* < 0.05), indicating that cooling with a cryomanchet led to a greater reduction in clinically rated tremor severity immediately after cooling than did cooling with a cold pack. For part B of the FTRS, a significant time effect was found (F(3.87) = 13.70; *p* < 0.0001). Post hoc tests revealed a significant decrease in tremor interference during functional tasks between the pre-measurement and all post-measurements (post-0, post-25′, and post-50′; *p* < 0.0001). The largest effect was seen immediately after cooling.

#### 3.1.3. NHPT

For the NHPT, for 10 patients who were able to complete the test at all test moments, a significant time effect was found (F(3.87) = 4.66; *p* < 0.05). Post hoc tests revealed a significant decrease in the time needed to execute the NHPT between the pre-measurement and immediately after cooling (post-0). Two patients could not complete the NHPT in the pre-condition, or at any other test moment (post-0′, post-25′, and post-50′). Two other patients could not complete the NHPT at the pre-condition but were able to complete the task at the post-0, post-25′, and post-50′ timepoints. One patient was not able to complete the NHPT in the pre-condition but could complete it immediately after cooling (post-0).

#### 3.1.4. TEMPA

**Figure 2:**
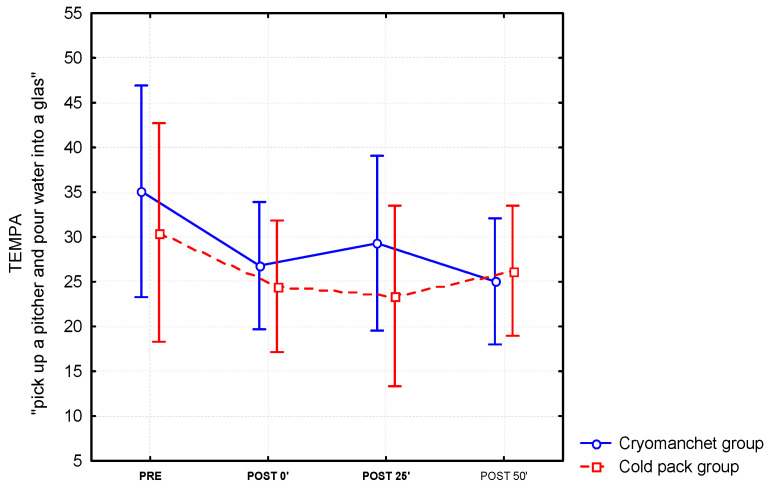
Differential effects of the cold pack and cryomanchet on the TEMPA “Pick up a pitcher and pour water into a glass” task (mean and standard error) for the forearm (*n* = 16).

For the timed scores on the unilateral TEMPA tasks, three items reached significant improvement after the application of a cold pack or a cryomanchet on the forearm. For the item “pick up a pitcher and pour water into a glass” (see Figure 2), a significant time effect (F(3.87) = 7.35; *p* < 0.0001) was found. Post hoc tests revealed a significant decrease in the time needed to execute the test between the pre-measurement and all the post-measurements(post-0, post-25 and post-50, *p* < 0.01). The main favorable effect was seen during the first 25 min after cooling (see Figure 1). For the items “handle coins” and “pick up and move small objects”, a significant time effect was found (F(3.87) = 4.91; *p* < 0.01 and (F(3.87) = 4.59; *p* < 0.01, respectively). Post hoc tests revealed a significant decrease in time needed to complete the tests between the pre-measurement and immediately after cooling (post-0, *p* < 0.05), and between the pre-measurements and after 50 min (post-50′, *p* < 0.01). A trend towards significance was also found between pre-measurements and after 25 min (post-25′; *p* < 0.06) for the item “pick up and move small objects”. Of the eight patients who could not perform the item “pick up a pitcher and pour water into a glass” of the TEMPA in the pre-condition, two patients were able to complete the task in the post-conditions, and one patient could perform this item with adaptation. 

### 3.2. Experiment 2: Cooling the Whole Arm

An overview of the results of temperature measurements and the clinical tests before (pre-) and after (post-0′, post-25′, and post-50′) whole arm cooling can be found in Table 3. 

#### 3.2.1. Skin Temperature

**Figure 3:**
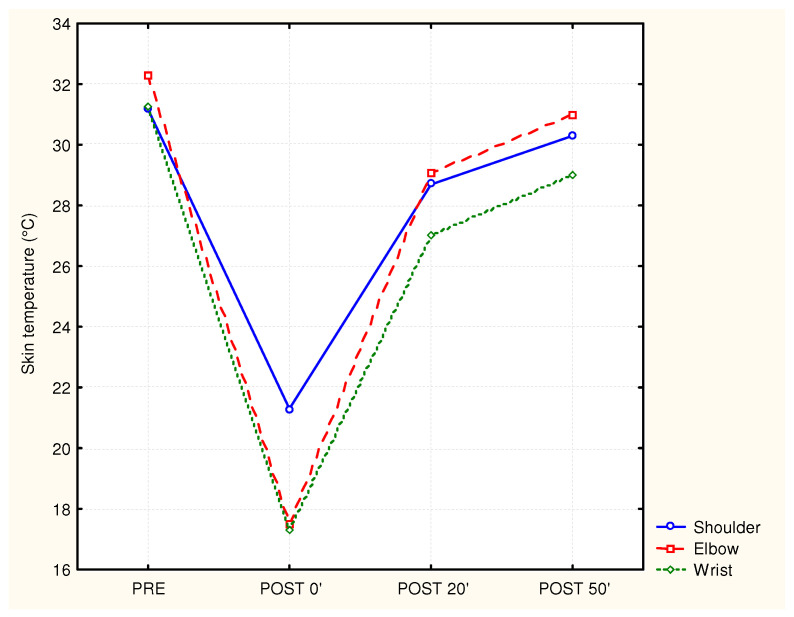
Mean skin temperature of the shoulder, elbow, and wrist before and after cooling (*n* = 17).

The skin temperature of the shoulder, elbow and wrist differed significantly between test moments, with F_shoulder_ (3.54) = 112.7 and *p* < 0.0001, F_elbow_ (3.54) = 166.6 and *p* < 0.0001, and F_wrist_ (3.54) = 116.4 and *p* < 0.0001, respectively. An immediate and significant reduction in skin temperature after cooling was seen at the shoulder (an average decrease of 11.3 °C), the elbow (an average decrease of 14.4 °C), and the wrist (an average decrease of 13.6 °C). After the removal of the cold packs, the temperature for all three of the arm positions increased in a curvilinear way with the highest warming up between the post-0′ and post-25′ timepoints. The temperatures at the post-50′ timepoint were still lower than the initial (pre) values. An illustration of the effect of cooling on the skin temperature at the shoulder, elbow, and wrist is shown in Figure 3.

#### 3.2.2. Handgrip Force and FTRS

The handgrip force did not significantly change after cooling the whole arm. Cooling the whole arm significantly reduced the scores in part A and part B, and the total score of the FTRS. The total FTRS score and part A of the FTRS significantly decreased (F(3.54) = 20.8; *p* < 0.0001 and F(3.54) = 13.3; *p* < 0.0001, respectively) immediately (post-0) and 25 min after cooling the whole arm (post-25′). For part B of the FTRS, the score significantly decreased (F(3.54) = 7.3; *p* < 0.001) immediately after cooling the whole arm (post-0). 

#### 3.2.3. NHPT

The time needed to complete the NHPT was not significantly different between test moments. Five patients could not complete the NHPT at the pre-condition, or at any other test moment (post-0′, post-25′ and post-50′). One patient could not complete the NHPT at the pre-condition but was able to complete the task at timepoints post-0, post-25 and post-50′.

#### 3.2.4. TEMPA

For the timed scores for the unilateral TEMPA tasks, only the two items “pick up and move a jar” (F(3.54) = 3.1; *p* < 0.05) and “pick up a pitcher and pour water into a glass” (F(3.54) = 4.2; *p* < 0.01) reached significance after the application of the cold packs on the arm. This reduction in performance time was observed immediately after cooling and remained significantly reduced for 25 min. Of the five patients who could not perform the item “pick up a pitcher and pour water into a glass” of the TEMPA in the pre-condition, one patient was able to complete the task at the post-0′ and post-25′ timepoints, and one patient was able to complete the task at all the post-conditions. Two patients were not able to complete the item “handle coins” in the pre-condition, or immediately after cooling. However, one patient could perform this task 25 min later and still had a better score in the post-50′ timepoint than before cooling. 

#### 3.2.5. Visual Analogue Scale

Cooling also significantly decreased the scores on the visual analogue scales. A significant decrease in the scores on both questions—i.e., “How difficult is it to complete the tasks?” and “How many tremor do you experience at this moment?”—was observed; F_VAS 1_ (3.54) = 5.30 with *p* < 0.01 and F_VAS 2_ (3.54) = 8.0 with *p* < 0.0001, respectively. This effect was only present immediately after cooling. After performing all tests immediately after cooling, the patients had to answer the question “Is your performance of these tasks better/worse than before cooling the arm?” by giving a score ranging from −3 (much worse) to 3 (much better). One patient answered that it was much worse than before cooling (−2), two patients said they noticed no difference (0), while ten patients said that it was better (1) and six patients even said that it was much better (2) than before cooling the whole arm.

#### 3.2.6. Sub-Analysis of Subgroups Based on Initial Tremor Severity 

**Figure 4:**
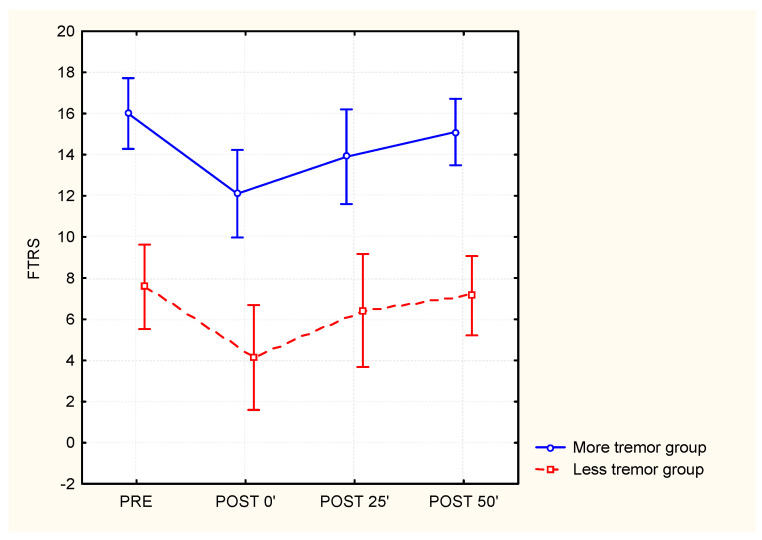
The effect of whole-arm cooling on the Fahn tremor rating scale for subgroups with different baseline tremor severities (‘less tremor’ group N = 8 versus ‘more tremor’ group N = 11 based on the median score on FTRS which was 12).

Further analyses were performed on subgroups with fewer or more tremors to investigate if the effects of cooling were dependent on the initial tremor severity. All illustration is shown in Figure 4 for the total FTRS score. No significant interaction between the subgroups and time points was found, indicating that cooling had a similar effect in both patients with mild and severe tremor.

## 4. Discussion 

The present study investigated whether or not cooling of the forearm or whole arm had effects on clinically rated tremor severity and functional performance in MS patients with intention tremor, and whether or not the effects were dependent on the cooling modality (active/passive cooling system) and application area (forearm/whole arm). In experiment 1, the forearm was cooled for 15 min with a cryomanchet or a cold pack in contrast with a control condition without cooling. In experiment 2, the whole arm was cooled for 15 min using multiple cold packs. Cooling the forearm (experiment 1) significantly reduced the FTRS, and the performance on the NHPT and three out of four items of the TEMPA. In experiment 2, cooling the whole arm reduced the FTRS and time needed to execute two out of four items of the TEMPA. These effects occurred immediately after cooling lasting at least 25 min. Cooling the whole upper limb led to a clinically noticeable effect on tremor severity and improved functional performance, which was pronounced during the first half-hour after cooling. 

Peripheral cooling was applied to the upper extremity with the hypothesis that a decrease in tremor severity intensity would lead to the improved motor control of the arm and enhanced functional performance. The hypothesis was based on an open-label study by Albrecht et al. (1998) [14] who reported improved functionality in MS patients with tremor after 1 min of immersion of the forearm in ice water, which persisted for 15 to 45 min after cooling. However, replicating pilot trials using ice water immersion was experienced as painful without major effects. Therefore, prolonged cooling techniques at more comfortable temperatures were investigated. A previous study showed that prolonged peripheral cooling of the forearm (for 15 min) using a cryomanchet connected to an active cooling device diminished the overall tremor amplitude, as measured during a one-dimensional step tracking task, for at least 30 min [17]. The effects of cooling on tremor severity seemed to be temperature-related, as the effects were dosage-related to the temperature of the applied cooling intervention. However, it remains unknown whether or not a cooling-induced reduction in tremor severity also leads to a decrease in tremor severity during unrestricted arm movements, and to improved hand/arm function.

The results of experiment 1 showed that cooling the forearm by applying a cold pack or a cryomanchet significantly reduced the clinically rated tremor severity (FTRS A), the total score on the FTRS, and the performance time of the functional tasks in the TEMPA and the NHPT. These effects persisted up to 25 min after cooling. Some patients, who could not perform an item of the TEMPA in the pre-condition, performed it independently in the post-condition. The sizes of the beneficial effects caused by forearm cooling using a passive (cold pack) and an active (cryomanchet) cooling system were further compared, as prompted by the clinical realization that the different cooling tools obviously have different costs. Direct comparison was possible as the same group of MS patients with intention tremor underwent both cooling modalities within a one-week interval. First, it appeared that the skin temperature of the forearm did not change significantly after a prolonged application of the cold pack and cryomanchet. However, with an active cooling system, a gradual, controlled decrease in coolant temperature could be applied while reducing the risk of frostbite or skin irritation. The main favorable different significant effect was found in the total score and in part A of the FTRS, indicating that cooling with a cryomanchet had a greater effect on the tremor severity than did cooling with a cold pack. A slightly different effect of cooling with a cold pack or cryomanchet was also found for the item “pick up a pitcher and pour water into a glass” of the TEMPA. The performance time at 50 min after cooling with a cold pack increased, while it still seemed to decrease after cooling with a cryomanchet. For the item “handle coins”, we found that the performance time at 25 min after cooling had a lower percentage with a cryomanchet than with a cold pack. 

Although beneficial effects were found after forearm cooling, one should be aware that these effects on the performance of functional tasks were rather small, i.e., approximately a 10% change, which is usually not regarded as clinically relevant. In fact, it was noticed that many participating patients seemed unconvinced of the clinical usefulness of forearm cooling due to the limited effect. The small effect may be explained by the following factors. The limited functional effect may be related to profound fine motricity deficits, since clumsiness in grasping objects was clinically observed in many patients. Even when the amplitude of hand tremor decreased after cooling, these patients still exhibited difficulties in performing tasks that required the fine motricity of the hand, such as picking up coins and small objects, although cooling had not influenced handgrip force. This may be related to the long-term disuse of the hand in fine motor tasks. Another explanation could be that forearm cooling was less effective in MS patients with a significant proximal tremor component, at improving the performance of functional activities that require movements in the proximal joints, such as the item “handle coins” or “pick up a pitcher and pour water into a glass”. The forearm was selected for cooling as the majority of MS patients exhibit a more pronounced distal tremor component compared to a proximal tremor component [19].

Since many daily life activities require multi-joint movements and that tremor is also common in the proximal joints, a second experiment was added sequentially to investigate the effects of whole arm cooling. In addition to the shoulder girdle and elbow muscles, whole arm cooling was also applied to the hand muscles. As hypothesized, cooling the whole arm via the application of multiple cold packs had a beneficial effect on tremor amplitude and the performance of functional activities and showed a significant decrease in the FTRS score after cooling. These effects were immediately observable after cooling, although limited in time. The performance times of the items “pick up a pitcher and pour water into a glass” and “pick up and move a jar” were significantly better immediately after cooling. In addition, some patients who could not completely perform an item of the TEMPA test in the pre-condition performed it independently in the post-condition. However, the NHPT and items “handle coins” and “pick up and move small objects” of the TEMPA, which are all fine motricity tasks, showed no improvement. This lack of improvement suggests that the severity of the tremor was still too great to facilitate fine motricity, but may also be indicative that profound deficits in manipulating fine objects are present in MS patients with tremor. This may be due to chronic disuse of the hand due to the permanent and longstanding presence of tremor during activity. Based on these results, one could conclude that cooling is more useful when trying to improve the functional performance of a mainly gross motricity task than that of a very fine motricity task. Improvement in the VAS score (i.e., VAS 1 and VAS 2) was also observed, while most patients reported an improvement on the Global Impression of Change scale. In summary, immediately after cooling, patients were generally satisfied with the effect cooling had on the severity of tremor and the functionality of their upper extremity. The effects of cooling on tremor severity or functional effects were similar in persons with different tremor severities, indicating its applicability to the spectrum of tremor severity. 

Another research question concerned the possible practice or fatigue effects due to the repeated performance of the functional tests. One could argue that a faster execution of the functional tasks after cooling could have been caused by the practice effect (i.e., more efficient performance of functional tasks due to repeated execution, rather than cooling). Conversely, the functional tasks may have been executed more slowly in the latter measurement moments due to fatigue after repeated executions during a relatively long test session of 1.5 h. To control for this, all tasks were also executed without any the application of any cooling modality. No significant performance differences were observed during the repeated executions of the control task, indicating that fatigue or practice factors did not significantly influence the performance. As such, it is concluded that the above-mentioned changes in the cooling conditions were directly caused by the cooling interventions. 

A clinically important issue is the duration of the cooling-induced effects on tremor severity and the functional performance of the upper limb. In the previous study of Feys et al. (2005) [16], the main finding was the marked reduction in overall tremor amplitude and frequency during the step tracking task after two different intensities (deep 18° and moderate 25°) after cooling the forearm with a cryomanchet for 15 min. Although the effects of cooling were probably temporary, they persisted during the full 30 min evaluation period. In our study, the performance of all timed scores on the unilateral TEMPA tasks had not reached their initial scores after 50 min. In the study of Albrecht et al. (1998) [14], in which the forearm including the hand was immersed in a bath of ice water for one minute, they also found that the scores had not reached their initial values after 45 min after cooling. Immersing the arm in ice water did not show an immediate effect on tremor, whereas our sustained cooling intervention did, probably because it was applied during a period long enough to affect the deeper structures of the arm. After some pilot trials, we considered cooling of the arm via immersion in ice water (with a temperature of about 4 °C) to be more painful and to come with a greater potential risk of freezing wounds in patients with impaired sensory function, than did the use of a cold pack or a cryomanchet. 

The effect of cooling has also been examined in other types of tremor, such as in patients with essential tremor (ET), which is an action tremor with postural and kinetic tremor. At a later stage of the disease, these patients may also develop an intention tremor component, similar to the intention tremor in MS patients [24]. Therefore, it seems justified to compare our results with studies involving patients with ET. In studies by Lakie et al. (1994) [25] and Cooper et al. (2000) [26], the hand or the forearm was immersed in a 15 °C water bath for 5 min. The cooling intervention led to an immediate reduction the in tremor frequency of the arm, as well as improved functionality as expressed in handwriting, archimedean spiral, drawing, card turning, simulated feeding, and stacking checkers. Post-cooling measurements showed that the effect, as measured in the study of Lakie et al. (1994) [25], remained present up to 30 min after cooling. Surprisingly, the functional improvements after our cooling intervention seem relatively similar to those in the immersion studies reported above, although the forearm or the whole arm was cooled for a longer duration and to much lower temperatures (reduction of 15.7 °C, 14.6 °C and 14.4 °C). 

### 4.1. Clinical Implication

It is concluded that peripheral cooling can serve as an effective treatment tool for inducing a temporary reduction in tremor while improving the functional activities of daily living, such as cooking, eating, writing, signing documents, and using a computer mouse. As a consequence, this may lead to a reduction in the patients’ dependency and improvement in their self-esteem. Possibly, not all patients would have the same effect of a specific cooling intervention because of the variety in the type and expression of the tremor. Cooling of the whole arm may be preferred because elbow, shoulder, and wrist control are also considered important for good functionality, and, in addition, tremor may also occur in the proximal joint. 

### 4.2. Study Limitations

The present study has some limitations. Two experiments were performed with different application areas of cooling, the being the forearm and the whole upper limb. However, a direct comparison of the effects was not performed given that the subjects in the samples were not identical, with the experiments being designed sequentially. Secondly, we applied functional tests and objective ratings of tremor severity but did not include quantitative measures such as digital spirography or kinematics of upper limb reaching movements [27]. Finally, we did not select patients based on tremor manifestation (distal versus proximal) or the purity of tremor presence versus ataxia [28]. The latter may have impacted the magnitude of the obtained effects.

## Figures and Tables

**Table 1:** Clinical characteristics of the patient groups in experiment 1 (N = 17) and 2 (N = 22).

Patient	Age (yrs)	Sex	Duration of MS (yrs)	Type of MS	EDSS	Postural Flexed *	Postural Extended *	Finger to Nose *	Pouring Water *	NHPT (s)
Experiment 1										
1	50	M	6	PP	6	2	2	1	1	56
2	48	F	11	SP	6	1	1	2	2	81
3	52	M	6	PP	6	1	2	3	4	137
4	39	M	3	RR	5	2	2	3	1	36
5	51	F	15	SP	6	2	1	1	1	51
6	42	M	6	SP	6	3	4	3	4	180
7	43	F	11	RR	8	1	2	2	2	148
8	40	F	12	PP	6	2	1	3	4	100
9	46	M	6	SP	5	2	3	3	3	85
10	33	F	6	RR	5.5	2	1	1	1	46
11	57	M	18	RR	5.5	2	1	2	2	46
12	55	M	39	PP	6.5	3	2	4	4	180
13	35	F	12	RR	7.5	1	1	1	1	31
14	43	F	11	RR	8	0	1	2	4	63
15	49	M	12	PP	6.5	1	1	2	2	58
16	51	M	26	SP	8	1	1	2	2	180
17	54	F	16	PP	6.5	3	1	3	4	120
Experiment 2										
1	52	M	7	PP	6	2	2	4	4	89
2	48	F	12	SP	6	1	0	2	2	70
3	49	M	14	PP	6.5	0	2	1	0	181
4	49	F	17	PP	6.5	2	2	3	4	300
5	40	F	13	PP	6	3	1	4	4	85
6	43	M	7	SP	6	2	2	4	4	300
7	49	M	13	PP	6.5	0	1	2	3	48
8	34	F	7	RR	5.5	2	1	3	0	59
9	52	F	16	SP	7	2	2	3	0	65
10	42	F	11	PP	7.5	2	3	3	4	300
11	42	M	17	RR	6	0	0	3	3	227
12	69	M	16	SP	6	1	0	2	2	92
13 **	56	F	32	SP	6.5	2	0	4	4	300
14	51	M	7	PP	6	1	1	2	0	49
15	63	F	26	PP	7	1	0	2	4	300
16	66	F	17	PP	6	2	2	3	4	116
17	46	F	14	SP	4	3	2	3	4	177
18	45	F	23	SP	6.5	2	1	3	3	39
19 **	53	M	26	PP	6	2	4	4	4	300
20	45	M	14	SP	7	2	2	3	4	300
21	55	M	15	PP	7	2	2	3	3	105
22 **	75	V	19	RR	8	4	4	4	4	300

PP = Primary Progressive; SP = Secondary Progressive; RR = Relapsing–Remitting; EDSS = Expanded Disability Status Scale (0–10); * Items from Fahn’s tremor rating scale (0–4); ** excluded for data analysis due to extremely severe tremor or fatigue.

**Table 2:** Overview of the results in the control condition, cold pack condition and cryomanchet condition in experiment 1 (N = 16). A two-way ANOVA was applied in the cooling conditions. Significant time effects are indicated in the table with ‘*’.

		Pre-		Post-0′		Post-25′		Post-50′	
		Mean	SD	Mean	SD	Mean	SD	Mean	SD
Temperature forearm (°C)	CP *	32.3	1.8	16.6	2.7	29	1.5	30.9	2
	CM *	32.3	1.3	17.7	1.1	28.3	2.3	31.1	2.1
Hand grip force (kg)	CO	20.4	10.1	19.9	9.9	19.9	9.8	20.1	9.6
	CP	21.1	7.7	21	7.5	22.3	8	22.5	8.7
	CM	21.8	8.5	21.7	8	21.8	7.7	22.4	8.4
Nine Hole peg test (s)	CO	78.8	37.2	67.6	36.3	83.5	42.7	81.6	42.2
	CP *	73.1	35.9	70.4	30.1	62.9	24.5	70.1	41.3
	CM *	82	40.7	72.9	34.9	78.4	41	78.1	43.9
TEMPA pick up and move a jar (s)	CO	3.7	1.1	3.6	0.9	3.5	1	3.2	0.8
	CP	3.4	0.9	3.5	0.8	3.3	1	3.3	1.1
	CM	4.7	4.7	4	2.9	3.7	2.3	3.7	2.6
TEMPA pick up a pitcher and pour water into a glass (s)	CO	32.4	18.2	29.6	14.9	30.4	19.8	25.9	13.2
	CP *	29.8	16.3	24	10.5	23.1	10.7	27.3	13.7
	CM *	35.1	27.8	26.8	16.4	30.3	25.7	24.6	15
TEMPA handle coins (s)	CO	27.4	14.8	26.7	15.9	27.7	18.5	22.3	8.2
	CP	32.3	27.1	29.2	23.1	28.3	23	27.1	23.1
	CM *	32.6	19.9	24.3	12.9	28.3	17.5	26.1	16.2
TEMPA pick up and move small objects (s)	CO	39.7	39.8	40.9	28.3	31.6	16.9	28.7	14.3
	CP	37.6	30	30.2	19.9	30.5	20.5	26.7	23.3
	CM	39.2	33.7	27.2	11.7	28.8	17.4	26.5	13.6
FTRS total	CO	14.1	5.3	8.9	5.7	8.7	6.3	7.1	5.8
	CP *	13.2	5.6	11	5.9	10.3	5.3	12.3	5.3
	CM *	14.1	4.9	8.6	4.6	11.4	5.6	10.9	3.9
FTRS part A	CO	7.5	2.3	3.5	3.6	3.8	3.8	3	3.5
	CP *	7.2	3.2	6.4	3.8	5.6	3.2	7.2	3.1
	CM *	7.8	2.1	5.4	2.8	6.3	2.7	7.3	2.5
FTRS part B	CO	6.5	3.4	5.5	3.7	4.9	3.8	4.1	3.7
	CP *	5.9	3.2	4.6	3	4.7	3.1	5	3.2
	CM *	6.3	3.5	4.6	2.8	5.2	3.6	5.2	3.4

* *p* < 0.05; FTRS = Fahn’s tremor rating scale; TEMPA = Test Evaluant les Membres supérieurs des Personnes Agées; CO=control condition; CP = coldpack condition; CM = cryomanchet condition.

**Table 3:** Overview of the temperature measurements and the clinical tests before and after whole arm cooling in experiment 2 (N = 22). A one-way ANOVA was applied with significant time effect indicated in the table by ‘*’.

	Pre-		Post-0′		Post-25′		Post-50′	
	Mean	SD	Mean	SD	Mean	SD	Mean	SD
Temperature shoulder (°C) **	32.1	1	20.8	3.6	28.6	2	30.2	1.6
Temperature elbow (°C) **	32.1	1.5	17.7	4.3	28.9	2.2	30.9	1.4
Temperature wrist (°C) **	31.2	1.6	17.6	4.6	26.9	2.9	28.9	2.6
Hand grip force (kg)	18	10.7	21.4	16	20.4	11.6	19.8	11
Nine hole peg test (s)	100	55.2	90	45.7	84.9	53.1	83.7	40.7
TEMPA pick up and move a jar (s) *	6.3	11	4.5	2.9	4.9	1.3	5.7	2.3
TEMPA pick up a pitcher and pour water into a glass (s) **	60.6	32.8	43.7	27.8	44.4	27.4	48.7	30.5
TEMPA handle coins (s) *	52.6	42.2	36.7	26.7	40.5	32.9	44.9	37
TEMPA pick up and move small objects (s)	47.4	42.1	44.9	51.4	44.7	40.4	42.2	29.1
VAS 1 **	7	3	4.5	2.3	5.5	2.6	6.2	2.7
VAS 2 **	6.6	3.3	4.2	2.9	5.3	3	6.2	2.9
FTRS total **	13.1	4.9	9.3	5	11.2	5	12.4	4.7
FTRS part A **	7.8	2.7	5.1	2.3	6.5	2.7	7.2	2.3
FTRS part B **	7.3	3.7	5.8	3.4	6.5	3.5	7	3.7

** *p* < 0.01; * *p* < 0.05; FTRS = Fahn’s tremor rating scale; VAS = Visual Analogue Scale; VAS 1 = “how difficult is it to complete the task?”; VAS 2 = “how many tremor do you experience at this moment?”; TEMPA = Test Evaluant les Membres supérieurs des Personnes Agées.

## Data Availability

Data can be made available upon request by the corresponding author.
